# The Human Microbiome and the Missing Heritability Problem

**DOI:** 10.3389/fgene.2017.00080

**Published:** 2017-06-13

**Authors:** Santiago Sandoval-Motta, Maximino Aldana, Esperanza Martínez-Romero, Alejandro Frank

**Affiliations:** ^1^Centro de Ciencias de la Complejidad, Universidad Nacional Autónoma de MéxicoMexico City, Mexico; ^2^Instituto de Ciencias Físicas, Universidad Nacional Autónoma de MéxicoMorelos, Mexico; ^3^Centro de Ciencias Genómicas, Universidad Nacional Autónoma de MéxicoMexico City, Mexico; ^4^Instituto de Ciencias Nucleares, Universidad Nacional Autónoma de MéxicoMexico City, Mexico; ^5^Member of El Colegio NacionalMexico City, Mexico

**Keywords:** missing heritability, GWAS (genome-wide association studies), MWAS (metagenome-wide association studies), microbiome, holobiont ecology

## Abstract

The “missing heritability” problem states that genetic variants in Genome-Wide Association Studies (GWAS) cannot completely explain the heritability of complex traits. Traditionally, the heritability of a phenotype is measured through familial studies using twins, siblings and other close relatives, making assumptions on the genetic similarities between them. When this heritability is compared to the one obtained through GWAS for the same traits, a substantial gap between both measurements arise with genome wide studies reporting significantly smaller values. Several mechanisms for this “missing heritability” have been proposed, such as epigenetics, epistasis, and sequencing depth. However, none of them are able to fully account for this gap in heritability. In this paper we provide evidence that suggests that in order for the phenotypic heritability of human traits to be broadly understood and accounted for, the compositional and functional diversity of the human microbiome must be taken into account. This hypothesis is based on several observations: (A) The composition of the human microbiome is associated with many important traits, including obesity, cancer, and neurological disorders. (B) Our microbiome encodes a second genome with nearly a 100 times more genes than the human genome, and this second genome may act as a rich source of genetic variation and phenotypic plasticity. (C) Human genotypes interact with the composition and structure of our microbiome, but cannot by themselves explain microbial variation. (D) Microbial genetic composition can be strongly influenced by the host's behavior, its environment or by vertical and horizontal transmissions from other hosts. Therefore, genetic similarities assumed in familial studies may cause overestimations of heritability values. We also propose a method that allows the compositional and functional diversity of our microbiome to be incorporated to genome wide association studies.

## Introduction: GWAS and the missing heritability problem

The *broad-sense heritability* (*H*^2^) of a phenotype is defined as the proportion of phenotypic variation that can be explained by genetic variance. A decade ago, genetic variance was almost impossible to measure accurately and was often assumed from kinship. Parents and offspring were assumed to have a 50% genetic identity between them, as with first siblings, whereas identical twins are assumed to have full identity. These studies are based mainly on pedigree data, so heritability estimates always included the contribution of all causal variants and several assumptions need to be made in order to calculate the heritability of a trait (Visscher et al., 2008). Nowadays, with the advent of Genome-Wide Association Studies (GWAS), estimates of the heritability of a trait can be based on the collection of Single Nucleotide Polymorphisms (SNPs) from populations of unrelated individuals. In order to estimate the narrow-sense heritability of a trait, these studies gather information of thousands of genetic variants and calculate the degree of relatedness between any two individuals through genetic identity. *The narrow sense heritability* (*h*^2^) is defined as the proportion of phenotypic variation that can be explained by genetic linear effects, and since GWAS associates individual SNPs it provides estimations of this type of heritability. As of today, we know more than 50,000 SNPs associated with many important human phenotypes. However, both individual and cumulative effects of these SNPs fall short of explaining the heritability of the phenotype they are associated with (Lee et al., 2011). For example, pedigree studies have shown that 80% of variation in human height comes from genetic effects. GWAS studies have found approximately 50 genetic variants that are associated with human height, but they are only able to explain 5% of height variation. This discrepancy between both measurements occurs in many human traits and is known as the *missing heritability problem*. Efforts aimed at finding its origin are still ongoing (Manolio et al., 2009; Eskin, 2015).

There are many possible explanations, and no consensus, as to where this *missing heritability* is hiding. Epigenetics, gene interactions, RNAs, heritability overestimations, small size effect variants, GWAS experimental limitations and many other factors have been proposed as possible reasons behind this problem (Slatkin, 2009; Marian, 2012; Zuk et al., 2012; Grandjean et al., 2013). Nevertheless, we are still unable to explain the complete heritability of human traits. In GWAS, an often reported issue arises when several SNPs are correlated with a given phenotype at a significant level, as these variants usually have small effect sizes. This means that, although many variants may be significantly associated with a single trait, having any one of them does not considerably increase the odds of developing the trait. One example is the LMTK2 variant in humans. Despite having a significant association with prostate cancer, the presence of this particular variant does not raise the odds of a person developing prostate cancer (Zuk et al., 2012). To circumvent some of these problems, updated versions of candidate gene studies have been proposed where instead of screening whole genomes, they choose to deep-sequence specific genes that have been previously identified through GWAS. This approach has a much higher resolution and has been useful for detecting new strong-effect variants (Zuk et al., 2014; Tsai et al., 2015). Nevertheless, these approaches still not provide a definite solution for the missing heritability problem.

In order to make this paper self-contained, in the sections below we first briefly discuss some of the major limitations of current genetic association studies and then review the current understanding of the microbiome and its importance to our physiology. Finally, based on these discussions, we propose as our main hypothesis that the existing gap between the heritability measured by GWAS on the one hand and familial studies on the other hand, can be significantly narrowed by taking into account the genetic and functional diversity of the microbiome, which is still a neglected source of phenotypic variation (Blanco-Gómez et al., 2016). We also provide a general perspective on how this calculations can be performed.

## GWAS limitations and gene interactions

One simplifying assumption often made by GWAS is that environmental factors such as behavior, diet and disease are homogeneous among their subjects. This assumption is usually not met. It has been shown that diet and exercise vary widely among groups of people and substantially impact the development of diseases such as obesity and diabetes (Pan et al., 1997). Habits may play an important role in the calculation of the heritability of these phenotypes, since people with similar behavior may be genetically distinct and yet express a close phenotypic resemblance.

GWAS also disregard epistasis (gene-gene interactions) and epigenetic effects. The study of gene regulatory networks has made clear that interactions across genes, proteins, RNA and other regulatory molecules are crucial to the generation and maintenance of specific gene expression patterns. These patterns, in turn, determine phenotypes. GWAS usually report individual SNPs associated with a specific trait, but SNPs can have combined effects that are not necessarily linear. A set of genetic variants can have synergistic or antagonistic effects when taken together (Wei et al., 2014). For instance, the co-occurrence of two SNPs could have a very strong positive effect which is not observed when only one of them is present, but if a third antagonistic variant is also present, the positive effect could be hindered, and the net result could be a mild association of the three genetic variants. Since interactions between sets of SNPs may also take place (McKinney and Pajewski, 2012), the development of statistical, mathematical and computational tools that can detect and take these schemes into account is crucial for determining the importance of epistatic effects. We should note, however, that from a simple probabilistic point of view, epistasis is often more relevant to physiology than to heritability, given the loss of gene correlations in offspring due to their parents' gene mixing (Young and Durbin, 2014).

Another source of phenotypic variation is epigenetic modifications. Although they have been mostly discarded due to the fact that almost all epigenetic marks are removed in the embryo through reprogramming, the question of whether epigenetic marks can be transmitted across human generations is still being debated (Slatkin, 2009). Methylation, acetylation, and mRNAs are known to influence gene expression (Delcuve et al., 2009), and this fact has critical consequences for the development of certain diseases. But unless these marks are preserved through several generations, their implication in the missing heritability problem cannot be significant. Since GWAS ignore these epigenetic marks, and we still do not know whether or not the epigenome is transmitted and to what extent, it is difficult to assess their role in the heritability of human traits. Nevertheless, there is a much large source of potential phenotypic variability that has not been taken into account when estimating the heritability of a trait.

## Humans as ecological adaptive systems

It has become increasingly apparent that the influence of the microbial communities living inside and over ourselves needs to be taken into account if we are to understand many aspects of our biology. There are about 3.9 × 10^13^ microbial cells inhabiting our bodies (Sender et al., 2016), and almost every part of it has a different composition and abundance of microbes (Morgan et al., 2013; Blekhman et al., 2015). These microbes interact with our metabolism in numerous ways and understanding the precise functionality and implications of these microbial organisms is a complex endeavor.

The influence of the microbiome on human biology has encouraged attention-grabbing statements in the literature, such as calling our microbiome an “additional organ”, or referring to the human body as a “super-organism” (Baquero and Nombela, 2012). However, these denominations can be misleading, as organs are composed of cells with the same genome, and a superorganism denotes an eusocial colony of individuals of the same species (Bordenstein et al., 2015). The term *holobiont* is more accurate and useful as it denotes a more dynamic entity composed of the genomes of many different species. Its composition can change in time and space as it adapts to new environments. Furthermore, the nuclear genome of eukaryotic cells is normally transmitted vertically trough a classic Mendelian framework, but the microbes of the holobiont can be passed on both vertically and horizontally. For example, specific symbiotic microbes, such as *Lactobacillus*, can be acquired through direct parental transfer, or by stable environmental transmission (Powell et al., 2014). Simple interactions with other humans, like kissing or touching, also lead to microbial transfers that can alter the composition of the *holobiont* (Kort et al., 2014). In the following section, we argue that ignoring the rich dynamics of the microbiome and our interaction with it can hinder our understanding of phenotypic diversity in humans.

## The influence of the microbiome in human traits

The microbiome has a strong impact in human health (Cho and Blaser, 2012; Clemente et al., 2012; Dave et al., 2012; Huttenhower et al., 2012). It is known that the human body has about the same number of bacterial cells as of human cells (Sender et al., 2016) and in terms of genetic content, the microbiome has at least a 100 times more genes than our own cells (Qin et al., 2010). The greatest diversity of microbial genetic content resides in our guts which have a preponderance of bacterial cells (~10^12^ bacterial cells per gram of colonic tissue Collins, 2014) and around 800–1,000 different bacterial species (Bäckhed et al., 2005). These findings have changed the traditional view of “one disease–one microbe” as many microbiome-related diseases are now thought of as a consequence of microbial community imbalances, better known as *dysbiosis*. This means that sickness arises not only from the presence of certain species, but also from their absence, relative abundance and/or interactions (Petersen and Round, 2014). For instance, microbial community disruptions are known to play a fundamental role in the progression of disorders such as irritable bowel disease (IBD), obesity and diabetes. Oppositely, high diversity and temporal stability of the gut microbiome are important characteristics of health. Detrimental states such as Crohn's disease and aging are commonly associated with a low diversity profile (Dicksved et al., 2008). There are, of course, examples of particular bacterial strains that are crucial to the progression of a disease such as *Helicobacter pylori* and the development of gastric cancer, but they are less common than previously thought (Perry et al., 2006; Spor et al., 2011).

One of the current major challenges in microbiome research is determining how essential the presence or absence of certain microbial species is to the development of a disease. Abundance of a species in the microbiome is not always an indicator of its importance in the development or presence of a trait. A seemingly innocuous initial imbalance in the microbiota can be further amplified via the action of specific pathogens that may have very low abundances. In periodontal inflammation, for example, it has been shown that certain keystone pathogens can magnify the virulence of the overall microbial community by disabling the immune response (Lamont et al., 2015). In addition to these innate pathogens, we know that certain bacterial species can suddenly become pathogens. These microbes, known as pathobionts, are relatively common in the normal microbiota, but given certain conditions, such as a mild loss of homeostasis in the host, they become amplifiers of imbalance. Through processes that promote inflammation in the host, or through the production of bacteriocins, these pathobionts can promote the pathogenicity of other strains in the microbial community, which naturally leads to further and stronger disruptions (Cho and Blaser, 2012).

## Shaping our microbiome

The interaction between microbiota and host is bidirectional, as the host also affects the development and stability of its microbiome. Firstly, changes in the host's diet and nutrition status can modify its microbial composition and behavior. Diets composed entirely of animal products are known to increase the abundance of bile-tolerant microorganisms like *Bilophila* and *Bacteroidetes*, whereas plant-based diets increase the abundance of *Firmicutes* that metabolize plant polysaccharides. These changes can be observed in the lapse of a week. In addition to changes in diet, exposure to antibiotics in food or clinical treatments can also have a fast and profound effect in the functioning and composition of the microbiome. It is known that the microbiome composition of an adult is relatively stable, but antibiotics, especially broad-spectrum, can kill entire communities of commensal microbes and also create a peak in the abundance of antibiotic-resistance genes (Yassour et al., 2016). A 5-day course of ciprofloxacin decreases overall bacterial diversity and changes the abundance of 30% of the species in the gut microbiome (Dethlefsen et al., 2008). Antibiotics equally affect non-pathogenic (commensal) microbes, often associated with the correct functioning of our metabolic processes and the development of our immune system. Their elimination can lead to an altered metabolism and to a malfunctioning immune response which in turn may induce additional imbalances in the microbiome. This can lead to pathogenic environments such as gut inflammation and an increased susceptibility to intestinal infections, as well as to further imbalances. Due to the interdependence of species, the removal of some strains (or specific functionalities) could also generate cascading effects in which microbes that were unaffected by the antibiotics themselves become extinct all the same, since they depend on the presence of other species. This interconnectedness is one reason behind the non-linear dynamics of the microbiome (Foster et al., 2008; Cho and Blaser, 2012).

Apart from their clinical use, antibiotics have several applications in cattle raising. Sub-lethal doses of antibiotics are commonly administered to livestock not only to prevent infections but also as growth enhancers. This observed weight increase in animals is now known to be related to a change in the metabolic capability and structure of the microbiome (Cho et al., 2012). Furthermore, antibiotic-resistant strains are strongly selected in these environments making multi-resistant pathogens like *Clostridium difficile* highly pervasive. In fact, infections by this pathogen are known to persist in human hosts even after the removal of the drug (Chang et al., 2008; Manges et al., 2010). Recent evidence shows that the backlash of antibiotic usage can last for weeks or even months after the removal of the antibiotic (Dethlefsen et al., 2008). According to meta-genomic studies, previously-undetected resistant genes appear in human fecal samples after a week of exposure to cefprozil (Raymond et al., 2016). Genetic horizontal transfers also play an important role in microbiome diversity as it can trigger a fast acquisition of resistant genes in many previously susceptible strains. It is thus not surprising that the overuse of antimicrobials in both clinical and agricultural contexts has been associated with the increased incidence of resistance strains worldwide (Raymond et al., 2016).

## Horizontal and vertical microbial transmissions

In 2013 *C. difficile* infections (CDI) were cataloged as an urgent threat in the report of antimicrobial resistance by the Centers for Disease Control and Prevention of the United States. CDI has a recurrence of about 15–30% and implies patient deconditioning, malnourishment and re-hospitalizations, among other inconveniences. Current treatments are based mainly on antibiotics and have a success rate between 30 and 80%, with recurrent infections being much harder to treat. However, transplants of whole microbial communities have shown great potential to control the growth of this pathogen (Khoruts et al., 2009). These microbial transplants (MT) consist in moving whole communities of microbes, typically from adult fecal samples, from one individual to another. By reintroducing possible missing species, this technique aims to restore the balance of the gut microbiome and diminish any dysbiosis that could have been promoted by the pathogen in the first place. As has been shown recently, after MT the microbiome composition of infected patients closely resembles that of healthy individuals (Weingarden et al., 2014) showing that MT can stably modify the microbial composition of the patients. Procedures using fecal MT have yielded success rates of nearly 100% in treating CDI with almost no side effects (van Nood et al., 2013; Li et al., 2016). This shows that a disease phenotype can be modified through the introduction of new microbes.

Another example of a microbiome-related disease is obesity, which affects more than a third of the worldwide population aged >20 years. This disease is a classic example of a complex trait to which genetics, diet, physical exercise and several other components contribute. Medical procedures that aim to treat these factors individually have shown a low success rate. Microbial transplants, however, have been proposed as a potential solution due to their high success rate observed with treating infections. Although, several studies have encountered differences in microbial phyla between obese and lean individuals, their relative proportions have not been consistently reported (Jayasinghe et al., 2016), making it difficult to propose a specific phylum responsible for obesity. In this sense, microbial changes associated with obesity probably have to do with subject-dependent microbial population structures, such as the relative abundances of certain species or functions, rather than with the presence or absence of a particular phylum (Walters et al., 2014). As of today, MT has not yet been approved for tests in humans to treat obesity. However, it has been reported that when a lean individual received the microbiota from an overweight person in order to treat a CD infection, the recipient experienced significant weight gain suggesting that human weight can also be modified by MT (Alang and Kelly, 2015).

MT in humans is now highly regulated by law and requires special permits to be implemented, so the vast majority of research with MT is done in mice. Specifically, germ-free (GF) mice are commonly used as recipients for microbial transplants. GF mice are a subset of a broad category of animals termed gnotobiotic, which include all animals that have a specific and known microbiota in them, including animals that lack any kind of microorganism. GF mice have proved to be excellent models for studying microbial transplants, because the microbial community of the host is controlled, and so are many other variables such as genetics, diet, and environment. It has been observed that the obese phenotype can be transmitted through microbial transplants in GF mice (Turnbaugh et al., 2006). GF mice that received microbial communities from obese mice exhibited a significant increase in body fat compared to GF mice that received MT from lean donors. Despite that microbial communities in mice differ from those in humans, similarities in gut microbiome between them have been found. One example is the proportion of *Bacteroidetes/Firmicutes* in obese as opposed to lean subjects, making it seem that these animals are good proxies for human gut studies. In fact, cross-species MT have been successful between humans and mice. After transplanting fecal microbial communities from an obese human to GF mice, human microbes were established and transmitted across generations of mice along with the obese phenotype, suggesting that important phenotypes can be transmitted across multiple generations through the microbiome (Turnbaugh et al., 2009b).

Apart from these examples of horizontal MT, there are natural and vertical ways to pass microbial communities from parents to offspring. The mode of delivery at birth, for example, has an important effect in the development of the child's microbiome (Dominguez-Bello et al., 2010). When meconium samples were analyzed for microbes, it was observed that babies born by cesarean section had a microbial composition similar to the one present in their mother's skin. In contrast, the microbiota of vaginally-born infants was more similar to that of the mother's vagina. Furthermore, the microbiome from cesarean-section newborns was associated with a greater risk of CDI, and a lower *Bacteroides* colonization which is linked to obesity. This same group of children displayed a 46% increase in obesity and a 20% higher risk of developing type 1 diabetes when compared to vaginally-born children (Cardwell et al., 2008). It has been hypothesized that initial bacteria play an important role in determining the establishment of other strains (Fanaro et al., 2007) and promote the proper establishment of the rest of the gut microbiome.

Breastfeeding is another vehicle of vertical microbial transmission in humans. Important microbial differences between formula-fed and breast-fed infants have been observed. Breast-fed neonates regularly show a higher abundance of the genus *Bifidobacteria*, which has been associated with health whereas formula-fed neonates have a lower microbial diversity which is commonly associated with disease (Koenig et al., 2011; Hermsen et al., 2012). In addition to the direct influence of the mouth-skin contact in breast-fed infants, the dietary components of human milk can influence the development of the child's microbiota. Important bioactive metabolites that are found in human milk can contribute to the development of the immune system as well as to the absorption and digestion of nutrients (Le Huërou-Luron et al., 2010; Horta and Victora, 2013). Milk oligosaccharides are the third largest component in human milk and are indigestible by humans. These compounds function as nutrients for intestinal microbes such as *Bifidobacterium*. Breast-fed infants that harbor higher abundances of these genera have been associated with the fortification of the gut mucosal barrier and exhibit a better immune response, improving their protection against pathogens (Liévin et al., 2000). Although delivery and feeding methods probably play the most important role, recent experiments suggest that the gut microbiota colonization may start while the baby is in the uterus (Jayasinghe et al., 2016). Although more tests are needed to establish which of these vertical microbial transmissions are more significant, it is important to note that vertical transmissions of microbiota occur, and have repercussions in the health of the offspring.

These experimental observations strongly suggest that there are several ways in which we can inherit microbes from our parents. However, the question of how this inheritance modifies the heritability of our phenotypes remains unanswered. When estimating the heritability of a phenotype, it is crucial to be able to separate genetic from environmental factors. As discussed above, the microbiome is involved in the development of several phenotypes, and its composition and genetic plasticity can change with environmental cues. This fact further complicates the separation of genetics and environment in any phenotype that has a microbial component. Nonetheless, heritability calculations that only consider human genetic variables may lead to incomplete results, as there is a close relationship between our genetics and our microbial communities.

## The genetic context of our microbial communities

The human body does not only provide a relatively stable and rich environment for its microbes, but also affects their composition and behavior. Gut microbial species and their abundances are being constantly controlled by the release of antimicrobial peptides in the intestinal epithelium. It is known that for the majority of human body parts, host genetics have been associated with the establishment and stability of the microbiome (Blekhman et al., 2015). For instance, some individual genes are known to greatly influence the diversity and structure of the gut microbiome, in addition to being associated with the progression of disease. The LEP gene, also known as OB or leptin-encoding gene represents a good example. This particular hormone acts as a cytokine and is known to control appetite, energy expenditure and other metabolic processes that alter our microbial composition. LEP secretion is directly linked to the amount of fat in the host, which is also associated with the proliferation of specific microbes. Mice with disrupted versions of the leptin gene (ob/ob) develop gut dysbiosis. In these animals the *Bacteroidetes* phylum has lower abundances when compared to normal mice (OB/OB) (Ley et al., 2005), and this change leads to an increased capacity for harvesting energy from diet, therefore increasing adiposity in the host (Turnbaugh et al., 2006). For additional examples of gene alterations that directly affect the composition of the microbiome see Spor et al. (2011).

Among all possible human genetic variation, the one occurring in genes related to the immune response has one of the highest potentials to influence microbial composition. Mutations occurring in genes directly involved in our immune system, such as those affecting immunoglobulins, HLA, or defensins, can change how we interact with our microbes—pathogens or otherwise. GWAS have provided techniques to examine the correlation between host genetics and microbiome traits. However, instead of analyzing specific genes and their effects on the microbiome, these studies have looked into human genetic diversity and its relation with the heterogeneity of microbial species, their relative taxa abundance and their functionality. By studying the genetic and microbial compositions of nearly 100 subjects, Blekhman et al. discovered that a positive correlation exists between similarity in genome sequences and similarity in the microbiome (Blekhman et al., 2015). In fact, in two thirds of the body sites tested, substantial associations arise between host genetics and microbiome composition, specifically in immune-related pathways. Interestingly, the associated human genomic regions showed high levels of differentiation, suggesting the host's genome adapts to specific environments and microbes.

Since our genetics influence our microbiome and vice versa, the fact that human-associated microbes and their respective abundances are highly personalized becomes more understandable. Nowadays, studying the less dominant taxa of a sample allows researchers not only to discriminate obese from lean individuals, but also to detect the part of the body where the sample came from, and even to discover the identity of the host within a group of individuals (Ursell et al., 2012). Around 10^6^ biological particles per hour are emitted from the human body to its surroundings. Therefore, apart from the common fecal sample analysis, most people can also be identified from their personal airborne release of microbes (Meadow et al., 2015). This result is, at first glance, at odds with the high variability of the microbiome throughout the development of a single individual, since the composition of the microbiome depends on diet and other environmental factors. However, it is precisely the characterization of abundances of rare microbial species—which may differ even if individuals live in a shared environment—that makes the personalization of the microbiome possible. This high personalization suggests a deep relationship between human genetics and microbial composition, supporting the idea that phenotypic resemblance within individuals may come not only from human genes, but also from microbial ones. Analogously, recent experiments have demonstrated that ethnicity and family bonds also correlate with the whole microbial composition. By analyzing fecal samples from Malawian and Venezuelan Amerindian families and comparing them to samples taken from the United States, Yatsunenko et al. discovered that microbial diversity was much more similar between geographically close individuals (Yatsunenko et al., 2012) than between individuals living in distant regions. Furthermore, early studies of the horizontal and familial transmission of bacterial strains in humans showed that specific strains of *H. pylori* were shared only between people that had frequent physical contact. In particular, *H. pylori* isolates were shared across nuclear family members in the United States, but not in South Africa. The authors suggest that this is probably due to the fact that physical contact in South Africa is more common between people without any kinship, in stark contrast to social norms in the United States (Schwarz et al., 2008). Taking into account the high rates of microbial interchange between our personalized clouds of microbes, physical interaction with strangers could be an important factor for the configuration of our microbiome. These results show that microbiome composition can be influenced not just by our genetics, but by our behavior and our surroundings as well, making it harder to separate environmental from genetic components. Since ethnicity and consanguinity are proxies for genetic similarity, microbiome-related phenotypes may appear to have a strong human-genetic component in familial studies if microbiome similarity is not taken into account, as ethnicity is related to habits and behaviors. The calculation of the heritability of a phenotype that is influenced by the microbiome can be strongly biased if the microbiome diversity is not taken into account, since microbial composition can be influenced by those common habits and behaviors. Thus, two individuals with a close genetic similarity due to ethnicity but different eating habits may display low phenotypic resemblance but high genetic similarity, which biases the calculation of the heritability of the phenotype in question.

In a nutshell, the microbiomes of related individuals are more similar than those of non-related individuals (Ursell et al., 2012). Microbial transfers can occur between individuals that have physical closeness or live in a similar environment, such as relatives in a family. Thus, if the development of a phenotype is related to the microbiome, looking only at single human genetic polymorphisms will not reveal the resemblance in genetic content and functionality between microbes belonging to different individuals. Consequently, a gap between phenotypic variance and observed genotypic variance will emerge with high estimates for the heritability measured in familiar clusters and low estimates in GWAS.

## GWAS and MWAS

Twin studies are an excellent source of information regarding the extent of the influence that our genome has over our microbiome. Possible human genetic influences on the microbiome might be far more easily understood in complex traits, like weight gain, when individuals are genetically identical. Weight gain is known to have a significant genetic component, but also has a strong microbial influence. Genetic variation and factors such as exercise and diet have been shown to influence weight gain. By studying this phenotype as a response to diet in twins, the similarity in phenotypes between twins (in the same pair) was about three times higher than among twin pairs (different pairs), suggesting that the stronger the genetic similarity between individuals, the lower the variation in phenotype (Bouchard et al., 1990). Nevertheless, this study only looks at human genetic identity, assuming it from kinship, but does not look at microbiome resemblance. Phenotypic similarity in identical twins may come from the fact that both have a more similar microbiome between them than among other twins (possibly due to similar environmental factors), and the observed resemblance in weight gain is not only due to genetic variation in human cells, but in microbial functionalities. Dietary bias, for instance, has a high correlation in twins. Food choices are significantly more alike between monozygotic twins (MZ; “identical twins”) than between dizygotic twins (DZ: “fraternal twins”), and a similar result was observed with responses to exercise (Savard and Bouchard, 1990). Despite the fact that the relationship between these factors and the microbiome is yet to be fully determined, it is well established that diet and exercise are directly related to weight gain, and also have an effect on our microbial composition (Spor et al., 2011; Kang et al., 2014).

Heritability studies often use MZ and DZ twins in order to have more control over both genetic and environmental factors. In such studies, heritability is usually measured by comparing the correlation level of a particular phenotypic trait between MZ and DZ pairs. Concretely, Falconer's formula states that the heritability of a trait can be measured by

$$h^{2}=2\left(R_{MZ}-R_{DZ}\right),$$

where *h*^2^ indicates the heritability of the phenotype and *R* is the correlation between each pair of twins. Consequently, if *R*_*MZ*_ > *R*_*DZ*_, the trait will have some degree of heritability, since the phenotype variation correlates with genetic identity. Early studies of the heritability of the microbiome found that there is greater similarity between the microbiota of MZ twins than that of DZ twins (which means that R_MZ_ > R_DZ_ for the microbiota, Zoetendal et al., 2001; Stewart et al., 2005) and this finding has been confirmed recently (Goodrich et al., 2014, 2016). Therefore, if the composition of the microbiota is considered as another phenotype, the fact that *R*_*MZ*_ > *R*_*DZ*_ for the microbiota indicates that there is a certain degree of heritability. Several microbial groups were observed to be heritable across generations, and human-microbial gene associations were also found, lending further credibility to the idea that our gene content influences our microbiome, and our microbiome influences our phenotypes. Hence, although many environmental factors play important roles in structuring the microbiome, genetic components seem to play a role too. It is worth noting that these studies look at the heritability of particular taxa or phyla of microbes, which as we discuss below, may not be as important as looking at their functions.

Controversially, there is another twin study that did not detect any heritable components of the microbiome (Turnbaugh et al., 2009a). In this study, the diversity of the fecal microbiome analyzed for each individual twin was measured through 16S rRNA sequences. The degree of similarity between MZ and DZ pairs was measured through the widely used UniFrac distance and their results showed no significant difference between *R*_*MZ*_ and *R*_*DZ*_, suggesting a lack of heritability of the microbiome. No phylotype was found to be present at an abundance of more than 0.5% in all samples, reinforcing their conclusion. As a matter of fact, when healthy populations were screened for shared microbial taxa, no particular taxa were observed to be universally present among individuals (Huttenhower et al., 2012). These results may suggest that the heritability of the microbiome is weak or circumstantial. However, it is important to remember that UniFrac distance does not account for the functionality of the microbiome, which can be different even between phylogenetically close individuals. UniFrac distance is a β-diversity measure (diversity between ecosystems), where the microbiome of two individuals will be more similar if the members of their microbial communities are phylogenetically close. In this sense, the functionality of the microbiome is assumed to be a consequence of phylogenetic resemblance. We now know that phylogenetic closeness does not necessarily imply similar functionality. In fact, a metagenomic approach that analyzed the gene content of the microbial communities in twins, uncovered many genes that were shared between them. However, they did not calculate the heritability of the microbiome using these data. Nonetheless, these results allowed for the identification of a “core microbiome” at the gene level instead of at the species level. It is therefore reasonable to think that it is the functionality of our microbes that is being strongly selected for. The functionality of the microbiome is not necessarily linked to specific taxa, as is often assumed in microbiome-disease associations. As previously discussed, the taxonomical diversity of the microbiome across individuals is very high, while the metabolic pathways remain relatively stable (Huttenhower et al., 2012). Even within the same genus, different strains can have very distinct metabolic pathways (Huttenhower et al., 2012). For instance, when the carriage of *Streptococcus* was measured in more than 200 individuals, the abundance of each *Streptococcus* species varied widely across them. Strain-level genomic variation and gene losses were very high, suggesting a link between the abundance of individual strains and the presence of different metabolic pathways. These events were related to host-specific genomic variants, which indirectly imply that the functional diversity of *Streptococcus* species within an individual will depend in part on the genetics of the host, and not so much on having specific strains of this bacteria. This suggests that the heritability of any microbial-associated trait is most likely to occur in terms of functional pathways as opposed to specific strains. Although circumstantial, the evidence presented so far strongly suggests that it is important to integrate the genotypic and functional diversity of our microbes into phenotypic association studies, and therefore into heritability estimates. In the section below, we point out some methodologies that incorporate the functional diversity of the microbiome into phenotype prediction.

## Heritability revisited through MWAS

Nowadays, the most advanced way to correlate human phenotypes with microbial genetic diversity is through Metagenome-Wide Association Studies (MWAS). These studies are largely based on the previously discussed GWAS, but instead of reporting individual SNPs associated with specific traits, MWAS measure the genetic diversity and composition of a complete microbiome sample and try to correlate it with a particular phenotype. One of the main advantages over ribosomal 16S studies is that MWAS provide enough resolution to look for enriched or impoverished metabolic pathways that could be involved in the presence of the phenotype at issue. MWAS can give a quantification of the abundance of the genes involved in each metabolic pathway, making it possible to identify bottlenecks in such pathways, or even missing links that can delay or prevent certain reactions from occurring.

MWAS, although expensive, is an undoubtedly powerful approach to analyze the way in which the microbiome can influence human phenotypes and to discover the mechanisms by which microbes interact with our bodies. Thanks to the Human Microbiome Project cheaper low-depth sequencing techniques are available that yield reasonable descriptions of human microbial samples. These techniques, such as shallow-shotgun sequencing, map the sequenced reads to previously-assembled genomes. With this mapping, researchers can infer, from fewer sequences, which organisms are present in their samples, as well as which genes and in what abundance. This correlation can also be computed by sequencing the transcriptome of the microbiome and then correlating the levels of the transcripts obtained with specific human genotypes. This type of correlations are commonly tested with expression quantitative trait loci (eQTLs), in which particular genotypes are correlated with the expression levels of a certain gene. Any of the above-mentioned methods is expected to outperform the widely used 16S studies in terms of the depth of information obtained, since they can describe the species of the sample as well as their abundances and functionality.

There are several important considerations that should be taken into account when trying to estimate the heritability of a phenotype through the inclusion of microbial data. First of all, despite that microbial composition may depend on our genetics to a certain degree, microbial genes are not ours. If we use the definition of heritability as the proportion of total phenotypic variance explained by the human genetic variance, microbes would be considered as external to our genetics. However, if we consider ourselves as an ecological system or *holobiont*, microbes and their genes are a significant part of it, and as we have discussed, they are capable of influencing our phenotypes. Thus, including their genes is only natural if we are to understand how heritable a phenotype is, and close the gap with familial studies estimations. Secondly, it has become apparent that microbial composition is strongly dependent on the environment. Since heritability is associated to genetic variance and not to environmental aspects, particular attention has to be paid to separate environmental factors from genetic ones when including microbial genetics. It appears that the microbiome blurs the line that separates genetic and environmental forces acting on phenotypes, as genetic components of the microbiome are much more flexible than our own and can change depending on external factors such as antibiotic ingestion, infections, dietary changes, etc. This flexibility makes the analysis more difficult, as taxa can change over a short time. However, gene content has proven to be a more consistent measurement in time and across individuals, to the point that a core microbiome can be defined in functional terms. This can prove useful when determining the microbial genetic factors that influence the development and heritability of a phenotype. Integrating data coming from GWAS and MWAS is not simple. GWAS data is categorical and each SNP has a discrete set of values. Values can be either 0, 1 or 2, depending if the genotype is homozygous for the major (0) or minor allele (2), or heterozygous (1). However, MWAS deals with continuous amounts. In fact, taxa abundance is not only continuous, but is also given in terms of relative abundance. Hence, careful normalization must be carried out before any valuable interpretation can be given. Microbial functional data is also given in terms of abundance, so each gene or gene cluster has a corresponding abundance whose correlation with the presence of the phenotype can be assessed. One possibility to incorporate microbial data into GWAS to estimate the heritability of traits, is to discretize the continuous variable into bins as is usually done in data mining techniques. This discretization may not be straightforward, as it may depend on the observed values in the population. However, once this step is achieved, several methods to estimate correlations with phenotype are already available (Lee et al., 2011; Bloom et al., 2013). If properly designed, twin studies can be of much use to control environmental and human genetic factors. For instance, follow-up studies that analyze the microbiome of twins in its infancy, when diet and behavioral habits are almost identical, can provide insights into how its composition is modified later in life, when such environmental factors are no longer similar. This may help explain to which extent the environment influences microbial composition, and how much of this variance correlates with the appearance of conditions such as obesity, diabetes, or several other gastrointestinal diseases. Finally, microbiome composition and functionality is known to change with age, gender and ethnicity, so other important factors to consider in the design of the study should involve population stratification. Calculating the heritability of a trait using GWAS and MWAS is certainly not a straightforward task and new statistical techniques will need to be developed. However, we believe that it is a worthwhile undertaking, since such methods will no doubt play an important role in the proper assessment of the role of our microbiome in our physiology and evolution, in the emerging paradigm of organisms seen as ecosystems.

Several techniques are available to calculate the heritability of a phenotype based on GWAS studies (Zaitlen and Kraft, 2012). As we show below, several of these techniques can be adapted to include microbial metagenomic data to estimate the narrow sense heritability of a particular phenotype. Mathematically, the narrow sense heritability is defined as

$$h^{2}=\;\frac{\sigma_{g}^{2}}{\sigma_{P}^{2}},$$

where $\sigma_{g}^{2}$ is the additive genetic variance, and $\sigma_{P}^{2}$ is the phenotypic variance defined as

$$\sigma_{P}^{2}=\sigma_{G}^{2}+\;\sigma_{E}^{2}+2\sigma_{G,E}^{2}.$$

In the last expression, $\sigma_{G}^{2}\;$ is the total genetic variance, $\sigma_{E}^{2}$ is the environmental variance, and $\sigma_{G,E}^{2}$ is the covariance between genetic and environmental components, which is often assumed as being nonexistent. In the additive model, phenotype of an j^th^ individual (y_*j*_) is defined by the sum of the lineal effects of its genetic variants:

$$y_{j}=m+\sum_{i\;\in \;C}z_{ij}*\alpha_{i}+\varepsilon_{j}.$$

In this equation *m* is a constant derived from the linear regression, *z*_*ij*_ are the normalized genotypes shown below (which will represent discretized abundances in MWAS), C is the collection of genotyped SNPs (genes or gene clusters in MWAS), α_*i*_ is the effect size for each variable and ε_*j*_ is the environmental contribuition. The normalized genotype *z*_*ij*_ is calculated as follows

$$z_{ij}=\;\frac{g_{ji\;\;}-2p_{i}}{\sqrt{2p_{i}\;(1-p_{i})}}\;.\;$$

When this formula is used to calculate the heritability of a phenotype using human genotypes obtained from GWAS, *g*_*ji*_ represents the *i*^th^ genotype of the *j*^th^ individual with values.

*g*_*ji*_ ∈ {0, 1, 2}, depending on the *j*^th^ individual being homozygous for the major allele (0), heterozygous (1), or homozygous for the minor allele (2) in the *i*^th^ SNP. Here *p*_*i*_ is the probability of the minor alleles in the *i*^th^ SNP. In GWAS the heritability of a trait is then calculated as the sum of the squared effect sizes for each normalized genotype (Zaitlen and Kraft, 2012):

$$\sigma_{g}^{2}=\;\sum_{i\;\in \;C}\alpha_{i}^{2}.$$

The effect size is often calculated through the Cohen's *D* parameter, which measures how big the effect of a particular SNP on the phenotype is.

However, the new metagenomic techniques allow us to calculate the effect size using abundances of particular microbial functions/genes instead of genotypes. This is so because MWAS report the abundance of all genes in the microbiome regardless of the particular organisms they belong to. These abundances must be discretized into *k*-values (or categories) for each gene in the population. After this categorization of each microbial gene/function for each individual, the effect size can then be calculated as

$$\alpha_{ik}=\;\frac{{\bar{x}}_{ik}-\mu }{\sigma_{ik}},$$

where ${\bar{x}}_{ik}$ is the mean phenotypic value of all the individuals pertaining to a category *k* of gene *i*, μ is the mean phenotypic value of the population, and σ_*ik*_ the phenotypic standard deviation. It is important to note that several definitions of the effect size exist depending on the selection of the standar deviation σ_*ik*_. For instance, Cohen's D uses

$$\sigma_{ik}=\sqrt{\frac{\left(n_{ik}-1\right)S_{ik}^{2}+\left({\hat{n}}_{ik}-1\right){\hat{S}}_{ik}^{2}}{n_{ik}+{\hat{n}}_{ik}-2}},$$

where *n*_*ik*_ is the number of individuals in the population that belong to the *k*^th^ category of the *i*^th^ gene and *S*_*ik*_ is the standard deviation of this quantity, whereas ${\hat{n}}_{ik\;}$ and Ŝ_*ik*_ are the corresponding quantities for individuals that do not belong to the *kth* category of the *i*^th^ gene.

With this effect sizes calculated for each category in each microbial gene, we can now compute the linear contribuition of the microbial genes to the heritability of the trait in question. Since the population phenotypic variance is normalized to 1, $\;\sigma_{P}^{2}=\;\sigma_{g}^{2}+\sigma_{E}^{2}=\;1,$ the microbial contribuition for the narrow sense heritability can be directly obtained as:

$$h_{MWAS}^{2}=\;\frac{\sigma_{g}^{2}}{\sigma_{g}^{2}+\sigma_{E}^{2}}=\sigma_{g}^{2},\;$$

where now $\sigma_{g}^{2}$ is the sum of all the $\alpha_{ik}^{2}$ computed using gene abundances. Since $h_{MWAS}^{2}$ estimates the linear contribuition of genetic microbial components to the heritability of a phenotype, it can be directly added to the heritability calculated through GWAS. We propose that the total narrow-sense heritability of a phenotype has to be computed as

$$h^{2}=\;h_{MWAS}^{2}+h_{GWAS}^{2}\;$$

## Conclusions and prospects

To summarize the ideas presented in this paper we consider human weight as an example of a trait where both human and microbial genetics are key factors to understand the missing heritability problem. The first estimates of the heritability of body mass index (BMI), taken from twins and family studies, were around 45%. More recent studies found heritability to be between 50 and 90% (Elks et al., 2012) but when GWAS was used to search for single nucleotide polymorphisms correlated with BMI, the ones found were only able to explain 2% of the phenotypic variation. This result clearly exemplifies the missing heritability problem, given the huge gap between the two measurements. Obesity is without a doubt a multifactorial disease that involves genetic and environmental factors. However, maternal obesity and diabetes are consistently among the most powerful predictors of childhood obesity (Redsell et al., 2013). A maternal Western diet has been shown to promote lipotoxicity and fatty liver disease in mice. In addition, it reduces the diversity of the intestinal microbiota which, along with lipotoxicity and fatty liver, persist in offspring, even after changing to a healthier diet. Note that it is particularly significant for our hypothesis that offspring display lack of microbial diversity too (Brumbaugh and Friedman, 2014). From these and other studies (some of which were discussed before) we conclude that the inheritance of such obesity-related treats cannot be attributed only to the transmission of the mice' genes. This in turn suggests that an initial imbalance caused by exposure to an obesity-related microbiota from overweight parents (breast feeding, uterus colonization or delivery mode) can have lingering consequences and can lead to the development of obesity in offspring. MWAS and 16S rRNA sequencing have shown a clear difference between obese and lean individuals in terms of species diversity and gene count (Le Chatelier et al., 2013). These observations strongly suggest that, when it comes to phenotypic variation in BMI, microbial composition plays an important role in determining weight gain. It follows that estimates of heritability of weight gain in familial studies may seriously overestimate the role of human genetics (heritability up to 90% compared to 2% in GWAS) and, in accordance to our hypothesis, probably such heritability arises from linear genetic effects of the microbes that are shared in such families. We suggest that most estimates of the heritability of BMI, and other human phenotypes, will be significantly improved if the similarity between microbial composition and function is taken into account.

To make this paper self-contained we first reviewed the role of the microbiota in physiology. We then presented evidence supporting the hypothesis that the microbiome must be considered in order to improve our estimates of the heritability of phenotypes. Our line of thought has been as follows. To begin with, GWAS have not been able to find the reported heritability obtained from twins and other familial studies. Even though epigenetics and epistasis have been proposed as alternative sources of phenotypic variability, no consensus has been reached regarding the causing mechanisms or the vehicle for heritability. Furthermore, no research so far has considered the microbiome and its functional and genetic potential in attempting to solve the missing heritability problem. It is crucial to take into account this enormous source of variability, as has been demonstrated by the large number of phenotypes it affects. Examples of this include the development of the immune system, obesity, diabetes, Crohn's disease, irritable bowel syndrome, cancer and neurological disorders. We have also emphasized in this paper the fact that our body can have a strong influence on the structure and composition of our microbiota. Diet modification and antibiotic usage are known to change our microbiome in fast, drastic and sometimes permanent ways. Additionally, metabolites released by our body can regulate the behavior of our microbial communities, which is an indicator of the ecological interconnectedness of the microbiome with human physiology. In terms of the inheritance of these microbes, it has been proved that both horizontal and vertical transmission between individuals take place, and that these transmissions are able to influence the receptor's phenotypes, such as making it more susceptible or resistant to disease. The nature of the particular microbes that are successfully transmitted between individuals may also depend on the genetics of the host. But since kinship or ethnicity are not only proxies for genetic identity, but also for behavior and diet (which may shape microbial composition), estimates of heritability based on family studies that do not consider the genetic variation of our microbes gives rise to the perception that phenotype variation is only due to human genetic resemblance. Since the correlation between microbiome composition and ethnicity or kinship could be caused by shared environments, similar diets, and other habits, studies with monozygotic and dizygotic twins that allow a certain degree of control over these variables must be developed. Some studies have shown only a slightly greater similarity between the microbiota of MZ twins than that of DZ twins, whereas other studies showed that no specific taxa were being inherited and that the microbiome varies by a very small amount. Nonetheless, when the functionalities of these microbiotas were considered, many shared genes were indeed found, allowing for the identification of a “core microbiome” at the functional level instead of at the species level.

We have suggested in this paper that when assessing the heritability of any human trait, searching for shared microbiome functionalities between the studied groups, instead of specific taxa, is of the utmost importance. Promising approaches to achieve this include combining GWAS and MWAS, data mining algorithms such as decision trees, Bayesian inference and supervised classificators, among others. Such an integration of the genetic diversity of the individual and its microbiome will most likely result in a more accurate calculation of the heritability for many important phenotypes, not only in humans but in livestock and plants as well.

## Author contributions

SS: Wrote the article, conception and structure of the article. MA: Wrote and revised the article, conception and structure of the article. EM: Critically revised the article, conception of the article. AF: Wrote and revised the article, conception of the article.

### Conflict of interest statement

The authors declare that the research was conducted in the absence of any commercial or financial relationships that could be construed as a potential conflict of interest.
